# KlugOculus: A Vision-Based Intelligent Architecture for Security System

**DOI:** 10.1155/2022/3722527

**Published:** 2022-05-17

**Authors:** Navjot Rathour, Rajesh Singh, Anita Gehlot, Neeraj Priyadarshi, Baseem Khan

**Affiliations:** ^1^Division of Research & Innovation, Uttaranchal University, Dehradun, Uttarakhand 248007, India; ^2^CTiF Global Capsule, Dept. of Business Development and Technology, Aarhus University, Herning 7400, Denmark; ^3^Department of Electrical and Computer Engineering, Hawassa University, Hawassa, Ethiopia

## Abstract

Vision-based system has gained significant attention in detecting the abnormal activities of intruders and alerting security with the amalgamation of adaptive video analytics techniques. The implementation of this kind of system works on face recognition, where the dedicated hardware with better computation power is limited in the previous studies. In this study, vision-based intelligent architecture and systems are proposed to detect intruders through facial recognition and sensors with customized hardware. As a part of the training, each subject was trained with 6 different pictures for a total of 120 images. Facial recognition implemented with machine learning (ML) inspired support vector machine (SVM) along with a histogram of oriented gradients (HOG). During the real-time implementation, the SVM model loaded in Raspberry Pi 3 has attained 99.9% accuracy for 20 different subjects. The proposed system can provide an accuracy of 99.9% even with tilted images of the subject, so it can be adopted by the different security personnel to boost the security system for the identification of intruders.

## 1. Introduction

Nowadays, security and safety are crucial elements due to the growth of crimes and thefts in the home. With the progress and advancement of technology, the home is embedded with distinct security systems and approaches to detect intruders [1]. Security and safety of the home are one of the applications of smart home automation with the Internet of Things (IoT). IoT enables interconnecting the distinct smart devices of the home to obtain real-time information from any location [2]. Surveillance systems have gained significant attention because of such issues that can be implemented for continuous monitoring and streaming of those videos live. In addition, ML techniques have also gained attention in classifying the real-time data precisely. Currently, along with IoT sensors, the camera-assisted system based on Raspberry Pi has gained wide attention for real-time monitoring through motion [3]. A Raspberry Pi and image processing-based system have been proposed to rescue isolated people, in which the camera-based Raspberry Pi mounted on a quadcopter streams the real-time video to rescue human beings [4]. However, they are a few limitations during the implementation of this system in real time for the detection and tracking of human beings. To resolve this issue, in this study we have proposed a system that integrates Raspberry Pi 3-based vision and a customized ATmega-based sensor for real-time detection of intruders through facial emotion. To implement this system in real time for the detection of a suspicious object, a trained model based on SVM and HOG is preloaded in the Raspberry Pi 3. The main contributions of the study are as follows:

- An architecture is proposed for the implementation of vision node-inspired real-time motion detection with Raspberry Pi-III and a customized ATmega328 board
- Raspberry Pi 3 is preloaded with a trained model based on a machine learning (ML)-inspired support vector machine (SVM) along with a histogram of oriented gradients (HOG)
- In the real-time implementation of the system, the model embedded in Raspberry Pi 3 has achieved an accuracy of 99.9% for 20 different subjects with accurate facial recognition

The structure of the article is as follows: Section 2 covers the literature review; Section 3 covers hardware description; Section 4 covers built and operation instructions; Section 5 covers validation and characterization, and the article concludes in the final section.

## 2. Literature Review

In this section, we present a previous literature review that is focused on enhancing the security system for the home with the assistance of wireless technology. Smart cameras play a major role in different areas of real-world applications, where a solar-powered smart camera named SlugCam has been proposed, to enhance the maintenance rate, and can be unattended for longer durations [5]. However, the environmental variation is a bit challenging in implementing smart cameras for video surveillance systems. To overcome these challenges, the IoT cloud server-based framework is implemented through an Internet protocol (IP) network, where the real-time variations like light intensity and background are rectified [6]. A smart monitoring system based on IoT has been proposed that can generate an alarm when motion is detected and it is connected to a cloud server via Raspberry Pi to send the photos and videos in real time [7]. The integration of Fisher's linear discrimination ratio for background removal has been incorporated in a study on the application of thermal video surveillance for the effective detection of moving persons [8]. A new algorithm is proposed with background subtraction and the Lucas–Kanade optical-flow algorithms to effectively classify humans and animals in an outdoor environment on video data along with pyroelectric infrared (PIR) sensors [9]. A new useful assistive system based on eye-tracking is proposed to control and monitor a smart home with IoT, where this assistive system helps a person with severe disabilities to control and remotely monitor everyday equipment in the home such as television, lamps, fan, radio, and the caregiver in real time [10].

A home security system against human intrusion using Raspberry Pi is presented in [11], and a camera motion estimation algorithm based on an optimized feature tracking method is applied for IoT devices. A study has utilized a Raspberry Pi-based system to recognize the emotions of the human through speech as speech recognition is effective in recognizing the emotions like anger, sadness, happiness, and disgust [12]. A real-time smart surveillance system based on Haar cascade and local binary patterns (LBP) has been proposed to monitor highly secured areas in real time, and it alerts the central monitoring authority via mail about the burglar if any change is found in real time via comparison of current frames with previous frames [13]. A vision-based home security system was implemented using OpenCV on the Raspberry Pi 3 Model B with the Haar cascade algorithm and HOG, and the findings demonstrate that the developed vision-based home security system has a higher detection rate than the PIR motion sensor-based security system [14]. Raspberry Pi-III and Arduino-based systems have been proposed that can record suspicious activities via a webcam that is mounted on the Raspberry Pi-III. HOG and SVM have been used for object detection purposes, and after the detection of an object, if some suspicious object has been detected, an alarm will automatically alert the owner to take corrective action with an efficiency of 89% [15]. In [16], the Raspberry Pi-assisted face recognition framework is presented for enhanced law-enforcement services in smart cities. A hybrid intrusion detection (HID) system with a machine learning algorithm is used to improve the security system by successfully evaluating the environment and user behavior to forecast future actions to secure the smart home system [17]. An intelligent security system based on content-based image retrieval is proposed for face recognition, where two different types of frontal facial images are analyzed and approximately 10,000 photos are collected from the police department offices, the Internet, and shootings for analysis [18]. A facial recognition system based on machine learning is proposed to detect an intruder in the home door locking system, where it enhances the security system with advanced techniques and effective accuracy [19]. An SVM is a very successful and widely used machine learning approach in the classification, object recognition, and image querying of vision-based security systems, where SVM was employed in conjunction with a range of feature extraction and face recognition algorithms [20].

From the above literature review and comparison in Table 1, the home security system based on the Raspberry Pi has gained wide attention in the detection of intruders based on motion. Few studies have implemented a speech recognition system to detect the emotion of humans for the security system, but due to noise environment and lack of effectiveness in concluding whether the intruder is known or unknown through their voice. So, Raspberry Pi 3-based security system with artificial intelligence (AI) model has wide priority for implementation of the home-based security system through vision data. Moreover, the customization of the hardware for real-time implementation is limited in the previous studies, and the integration of sensors and the vision-based system is also limited in the previous studies. This study implemented customization hardware for the implementation of a sensor-based system along with an SVM-loaded Raspberry Pi 3. The proposed system achieved an accuracy of 99.9% for facial recognition during the motion with titled images.

## 3. Hardware Description

KlugOculus is a customized board that has been designed to implement fast processing in real time with a minimum power requirement. KlugOculus is designed with readily available components on the Raspberry Pi and Arduino UNO boards. KlugOculus is powered up by a power supply that has been designed to give continuous supply to the board. Depending upon the application, KlugOculus can be powered up via solar cells that can be recharged via sunlight when discharged. KlugOculus has wider capabilities for processing. It can process videos in real time via artificial intelligence (AI) and ML algorithms. The Pi camera is used to record the videos in real time. The Pi-Cam is normally turned off and will only be activated when the PIR sensor, which is connected to the Arduino board, sends a signal to the Raspberry Pi. Pi-Cam will record the video in real time and save the recorded video on a cloud as well. The recorded video can be used in the future for further investigation or extraction, depending on the nature of the application. The main design goals of KlugOculus are mentioned as follows:

### 3.1. Cost-Effective

KlugOculus is unique because it is designed using two different units, and both of those units are low-power-consuming units. The Arduino will consume power when an intruder is detected by the PIR sensor, and the moment Arduino is in action, the Raspberry Pi remains in an off condition. This can be achieved by using a smart trip wiring system while connecting these units with a power source. After detecting an intruder, Arduino will turn off, and Raspberry Pi will handle further processing via Pi-Cam and real-time video streaming, as well as saving the recorded video to the cloud. This smart switching and the availability of alternate power sources like solar-enabled power systems will make KlugOculus a cost-effective system.

### 3.2. Diversity

The main purpose behind the design of KlugOculus is to provide a compact, wireless, and powerful system which can be used easily for wired applications. KlugOculus powered by solar cells or battery banks will make it possible. The system does not need to be connected to wires or power sources all the time.

### 3.3. Extensible and Open Source

Another major goal of designing KlugOculus is to provide a platform for the research community. To do that, we have kept the design simple, and both the platforms are open source. At present, we have used Arduino for connecting the PIR and Raspberry Pi for connecting the Pi camera. In the future, this can be further extended by using various sensors. The use of open-source hardware and software has made it more flexible for further modification and use.

**Figure 1:**
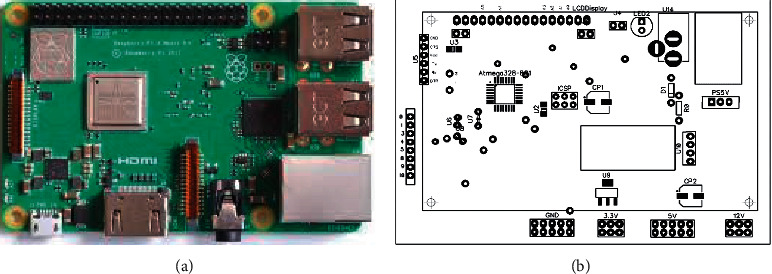
(a) Raspberry Pi 3B+ Model. (b) Bit map of ATmega328 customized board.

**Figure 2:**
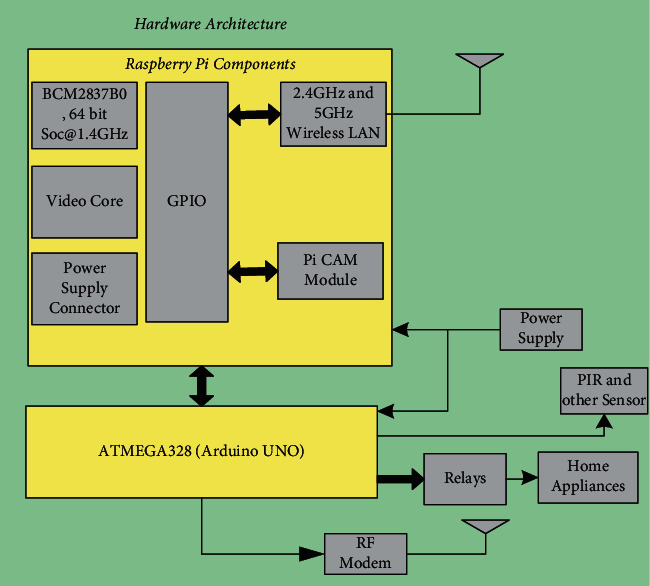
KlugOculus hardware architecture.

Figure 1 shows the Raspberry Pi 3B+ Model and the ATmega328 customized board. This model comes with an in-built Wi-Fi module that will help to connect KlugOculus with the cloud. The Pi camera module helps to capture images and videos in real time. The hardware architecture of KlugOculus has been represented in Figure 2. The hardware architecture is mainly divided into two major sections. One unit is a Raspberry Pi, and another unit involves an ATmega328. The Raspberry Pi unit connects the camera module and helps KlugOculus transmit captured data to the cloud with the help of the onboard Wi-Fi module.

**Figure 3:**
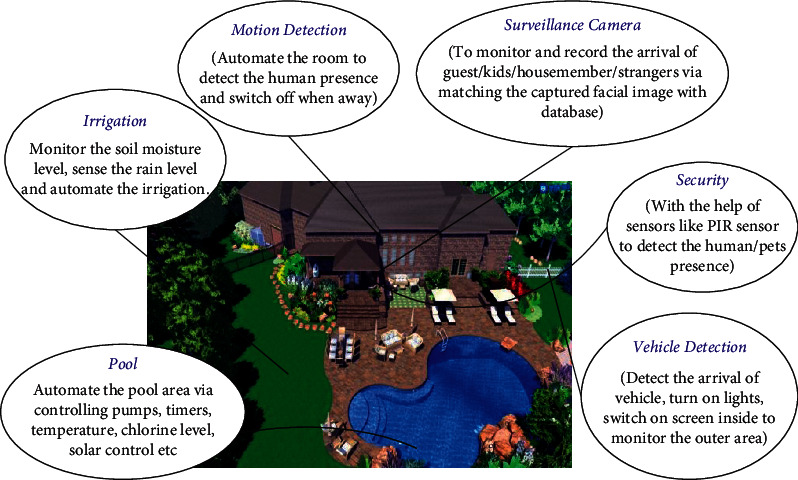
Integrated home automation.

Various sensors that can be used in the entire system are interfaced with the help of ATmega328. The Arduino board interfaces with the Raspberry Pi using pyFirmata. Sensors that are used to detect the presence of human beings, like PIR sensors, are interfaced with the ATmega328. The Arduino UNO is also provided with an RF modem that will help with multinode communication. Figure 2 shows the proposed system of home automation. It covers various aspects that will help to implement home automation using KlugOculus (Figure 3). With the help of multiple nodes, devices can communicate with each other. For example, the surveillance camera is recording images or videos of human beings at the main door of the home, and after detection via PIR sensor, the video can be live streamed and displayed on the monitor inside the room. This will help to authenticate the person at the main door and will help to increase safety and security.

**Figure 4:**
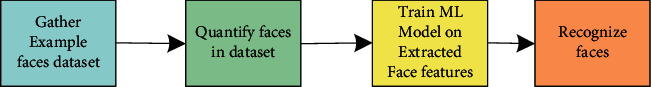
Flowchart to build a face recognition system.

The proposed system is also making our homes energy efficient as PIR sensors installed inside the room will help to detect the presence and movement of human beings inside the room and will automatically power off the lights and fans when no movement is detected. Similarly, the arrival and departure of vehicles will also turn on and turn off parking lights automatically, and the surveillance camera will send the recorded video of the vehicle to the cloud and live to stream it also. This will again help to ensure the safety and security of the home. Not only can this be done, but automation can also be done with the help of KlugOculus nodes installed in the pool area and garden area as well. The pool area can be automated by automating the pumps, timers, water temperature, chlorine level, solar control, etc. Furthermore, soil moisture-level monitoring and automation of the soil irrigation system will aid in the achievement of trouble-free maintenance in subsequent outdoor areas of the home. The flowchart shown in Figure 4 shows the basic flow of the face recognition system. Primary requirement to build the system is to gather the dataset images. Dataset images should be of standard resolution that can be trained on the system. After building the dataset, the following two steps need to be followed:

- Quantification of each face using Dlib and face_recognition libraries
- Train a support vector machine (SVM) on the face quantification

Once the above two steps are complete, our system is ready for making the predictions.

### 3.4. Software Architecture of Raspberry Pi Manager

**Figure 5:**
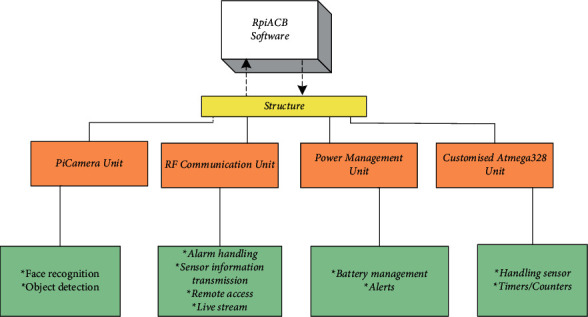
Software architecture.

The Raspberry Pi works on the Linux platform, which is a powerful platform. The customized distribution makes this system more powerful to work with. The software architecture of the Raspberry Pi manager is mainly divided into 4 units, named the customized ATmega328 unit, power management unit, RF communication unit, and Pi camera unit as shown in Figure 5. All these units work on all the “*n*” nodes as shown in Figure 5.

### 3.5. Customized ATmega Unit

This unit will manage the sensors that are being interfaced to sense various inputs and also help to manage these sensors in real time with the help of timers and counters.

### 3.6. Power Management Unit

The power management unit will take care of battery levels and generate all the alerts when the battery reaches a critical level. This unit will manage the power and handle critical situations related to the battery.

### 3.7. RF Communication Module

**Figure 6:**
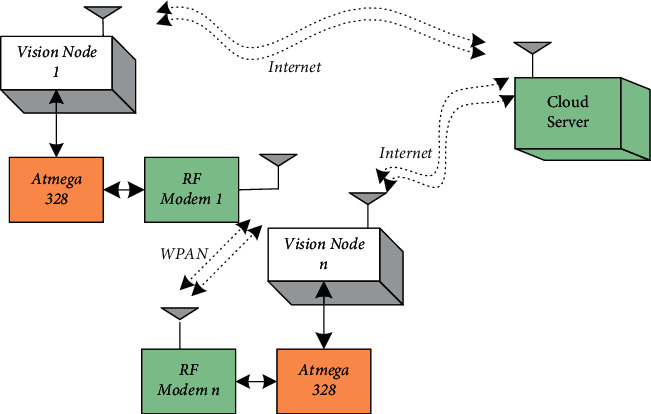
Multinode deployment of KlugOculus.

The RF communication unit is another unit that handles all the sensors interfaced with the ATmega328 unit. This unit will help in gathering the data from the sensor unit and will help in the live streaming of this data. Inter-domain communication becomes possible with the help of this particular unit. As shown in Figure 6, “*n*” vision nodes can communicate with each other with the help of these units. Moreover, such units provide a wide scope of remote access which is the utmost requirement of the majority of systems.

### 3.8. Pi Camera Unit

**Figure 7:**
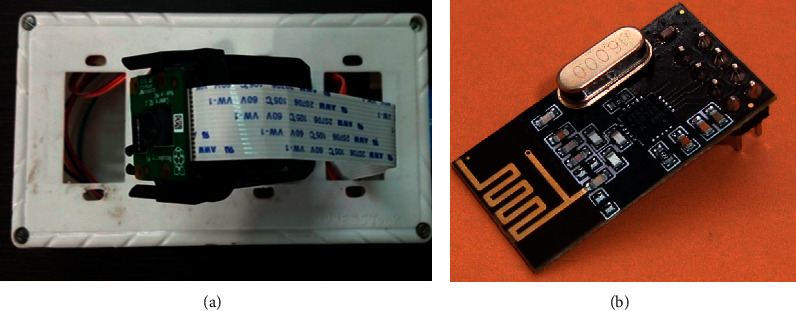
(a) Vision node. (b) RF modem.

This unit is mainly important because it handles the critical task of objects and faces detection. This unit mainly captures the images and records the videos of human beings and objects. This unit captures the facial images and, with the help of OpenCV, detects the faces in real time using various machine learning algorithms. Object detection is also carried out to automate the system along with sensors. This unit works according to the signals sent by sensors like the PIR sensor so that the camera unit will enter into sleep mode and save power, and will enter into wakeup mode once signals have been sent by the sensor, to begin with, object detection or face detection.  Figure 7 shows the vision node of KlugOculus and RF modem for the same.

## 4. Build and Operation Instructions

**Figure 8:**
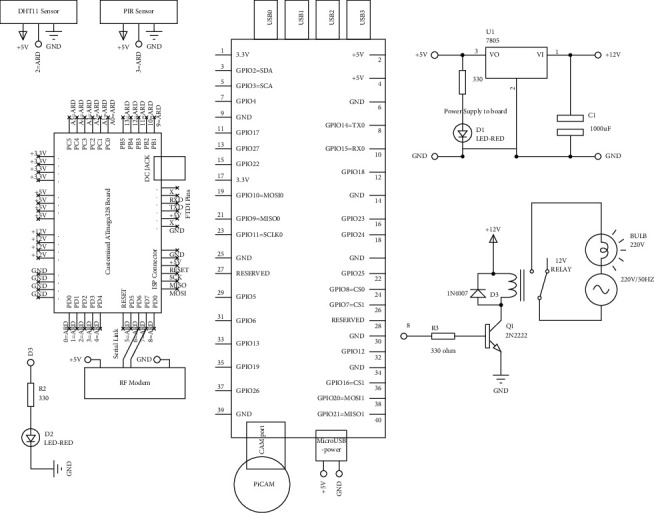
Circuit diagram of KlugOculus.

The circuit diagram of the proposed system is shown in Figure 8. A detailed pin description along with the connections on a particular pin has been represented. The RF modem is connected to a customized ATmega328 board at PINs 6 and 7 to read the analog values. The PIR and DHT11 sensors have been connected to PINs 2 and 3. The Pi-Cam has been connected to capture the data in real time with the help of the camera port available on the Raspberry Pi. Relay has been connected to control the devices and automate the room. Pin Number 8 has been connected to the relay. The power supply used for the circuit has also been shown.

### 4.1. Cloud Architecture

**Figure 9:**
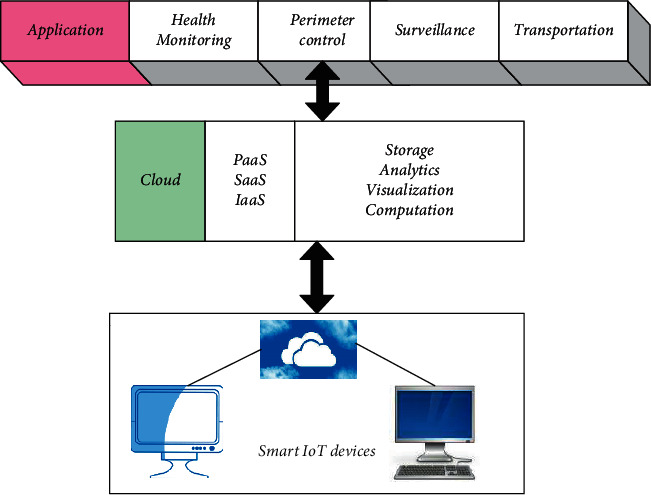
Basic cloud architecture.

As per the services selected for the cloud, it will provide users with a cloud environment that will help them to manage, develop, and deliver applications.  Figure 9 shows the cloud architecture. Depending upon the services opted for by the user, various applications have been categorized. A few applications have been mentioned in Figure 9 as health monitoring, perimeter control, surveillance, and transportation.

**Figure 10:**
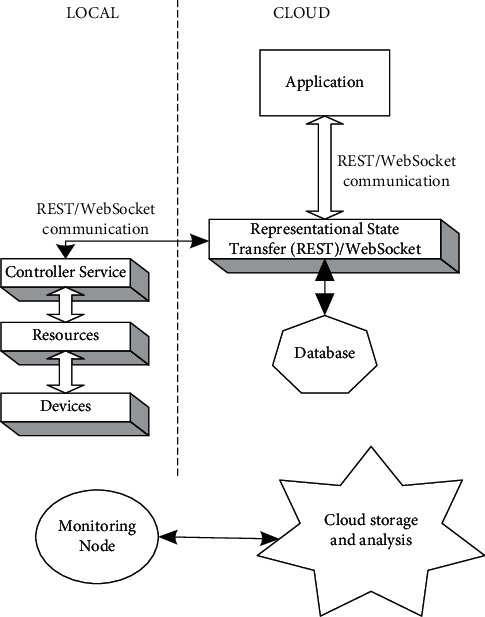
“Internet of Things (IoT)” Level 3 systems.

Figure 10 shows the Level 2 of “Internet of Things (IoT)” in which the data are stored directly on the cloud. Such an arrangement detects and senses the data and stores it in the cloud. This level of “Internet of Things (IoT)” is used when the data are in large amounts. The Level 2 architecture will have only a single node, i.e., the vision node in this application. The sensory node will detect the data in the real time and live stream it on the cloud. The database of facial images is already available for which the computational part is carried out directly on the cloud. As shown in Figure 10, the final application is to recognize faces and the protocols used for this are REST and WebSocket. REST is one of the most reliable ways in which API requests are structured, and WebSocket is also a standardized protocol via which two-way communications over a single TCP connection are established.

**Figure 11:**
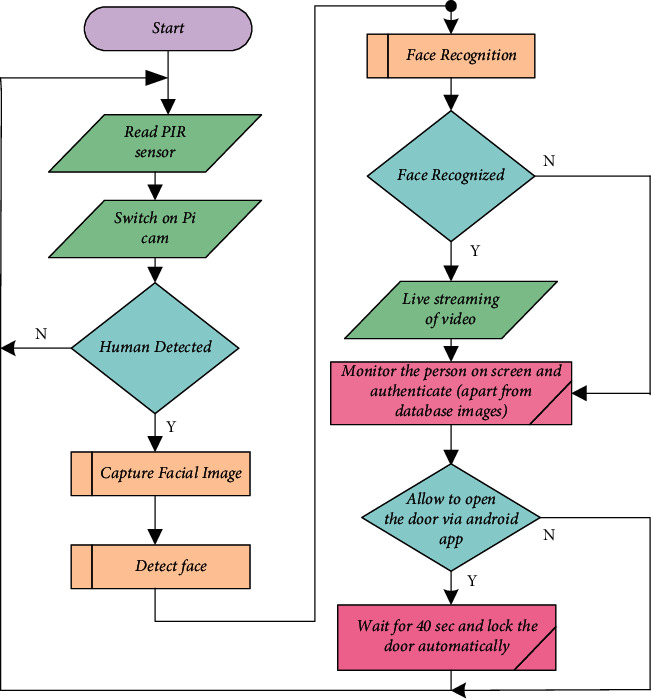
Flowchart of vision node.

Figure 11 shows the flowchart of the vision node which is capable of taking the decision of opening and closing the door as per the authentication provided by the owner of the house. If the person at the door is already available in the database, then the person will be allowed only after permission is granted by the owner. The door will remain closed if permission is not granted by the owner of the house. Moreover, the entire node will come alive only when the PIR sensor activates the Pi-Cam after the detection of a human being. The entire system will provide two-tiered security and safety to the home from suspects and intruders. The major role here is being played by the face detection algorithm that is being run on the Raspberry Pi.

## 5. Validation and Characterization

Recognition of faces consists of multiple iterations. A large number of algorithms are available to recognize human faces. Face recognition in real time encounters a plethora of challenges when it comes to hardware implementation. Facial recognition on the Raspberry Pi encounters numerous challenges because of its limited capability and processing speed. With the help of OpenCV, Python, and Dlib, it becomes easy and effective. There are multiple steps included in the detection and recognition of facial images.

**Figure 12:**
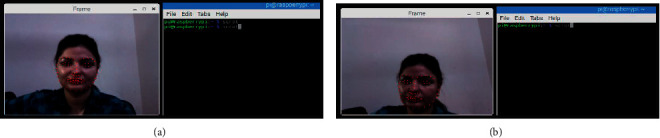
Facial landmark detection. (a) Facial landmark detection with frontal face. (b) Facial landmark detection with tilted images.

**Figure 13:**
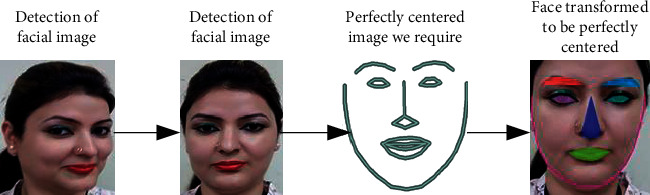
Image transformation using facial landmarks.

**Figure 14:**
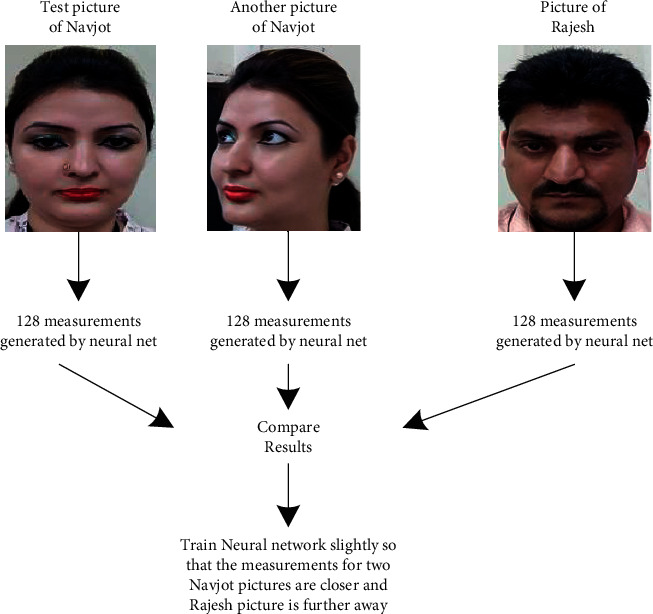
Generation of 128-dimensional data from triplet.

**Figure 15:**
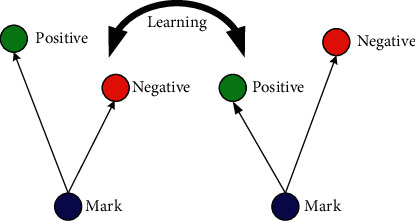
Triplet loss.

**Figure 16:**
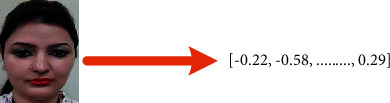
Generation of 128-d real-valued number feature vector of each training image in the dataset.

**Figure 17:**
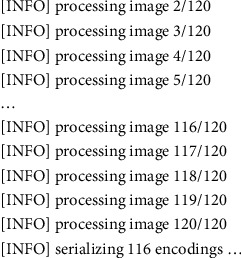
Computation of facial embeddings with OpenCV and Movidius.

Step 1: The very first step is the detection of faces in real time. Face detection became popular in the early 2000s when the most recognized algorithm was named after its developers, the Viola–Jones algorithm. However, a humungous number of methods are available, out of which the well-known method called histogram of oriented gradients (HOG) has been used. HOG converts the colored image to a black and white image as colored data are of no use to locate faces. To locate the facial image, gradients with the strongest values have been located, and after the placement of the strongest gradients on the HOG image, the facial image is located by comparing it with features of the most similar pattern. These HOG patterns are collected via the use of several other training images.
 Step 2: After locating the face, the next important step is to pose and project the face. As tilted or turned, the face looks different from the computer (Figure 12). So, to resolve this issue, the face landmark estimation algorithm has been used. This will help to trace 68 facial landmarks, which are specific points that exist on each individual's face as shown in Figure 12. After locating these points, a training algorithm has been implemented so that these 68 points can be located on any face. Once all the features like eyes, mouth, nose, lips, eyebrows, etc., are located, basic image-related transformations are applied to scale and rotate the image to make it recognized by the machine in a better way, as shown in Figure 13.
 Step 3: The final step is to tell the two faces apart. In order to achieve this with accuracy, the system needs to be trained. The most efficient results can be achieved by measuring the faces and letting the system decide using deep learning, as a machine can decide better which part of the face is important and which is not. A deep network measures the 128 measurements for each face, and instead of a single face, the network has been trained on 3 facial images at an instant, as shown in Figure 14. This is achieved by training the first image (image mark) of a person with a second image (positive) of the same person with a completely different image (image negative) of other people. The main purpose is to have the image mark closer to image positive as compared to any other image called image negative. The selection of triplets to carry out the 128 measurements is important. Machine learning people call these measurements of every individual face an embedding. Training on face embedding using a huge set of images called a dataset will improve the accuracy and decrease the error rate eventually.
 This process requires huge CPU power and a lot of time. To understand triplet loss, consider the representation as ‖*f*(*y*)‖ ∈ *ℝ*^s^ which is representing and image *y* into s-dimensional Euclidean space. We oblige this implanting to live on the s-dimensional hypersphere. ‖*f*(*y*)‖_2_ = 1. As shown in Figure 15, the main aim is to achieve a minimum distance between *y*_*j*_^*m*^ (mark) of a specific person with all the other images *y*_*j*_^*p*^ (positive) of the same person as compared to the image of any other person *y*_*i*_^*n*^ (negative).
 So, we want to have
  (1) $$\begin{array}{c} {\left\|y_{j}^{m}-y_{j}^{p}\right\|}_{2}^{2}+{\left\|\beta y_{j}^{m}-y_{j}^{n}\right\|}_{2}^{2}\forall \left(y_{j}^{m},y_{j}^{n},y_{j}^{p}\right)\in \zeta , \end{array}$$
   where *β* is the enforced margin between negative and positive pairs of images and *ζ* is the set of all the possible triplets and has numbers equal to number *M*.
  (2) $$\begin{array}{c} \overset{P}{\underset{j}{\sum }}{\left[{\left\|f{\left(y\right)}_{j}^{m}-f{\left(y\right)}_{j}^{p}\right\|}_{2}^{2}-{\left\|f\left(y_{j}^{m}\right)-f\left(y_{j}^{n}\right)\right\|}_{2}^{2}+\beta \right]}_{+}. \end{array}$$
 The generation of multiple triplets will help to overcome the issue faced in equation (1), and the selection of suitable and complex triplets will result in the improvement of the deep learning model.
 Step 4: The last step is identifying the person, also known as classification. Varieties of classifiers are available. Linear SVM is the most common and efficient classifier. All the classifiers can work, but the main aim is to train the classifier, so that it recognizes and gives the result in the form of the closest match. The final result of classification is the name of the person as shown in Figure 16. The computation embeddings are presented in Figure 17.

**Figure 18:**
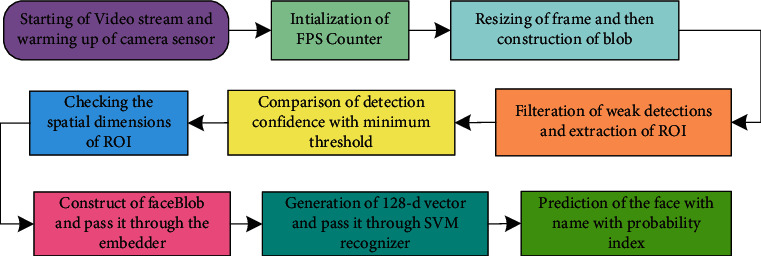
Process flow of face recognition in real time.

**Figure 19:**
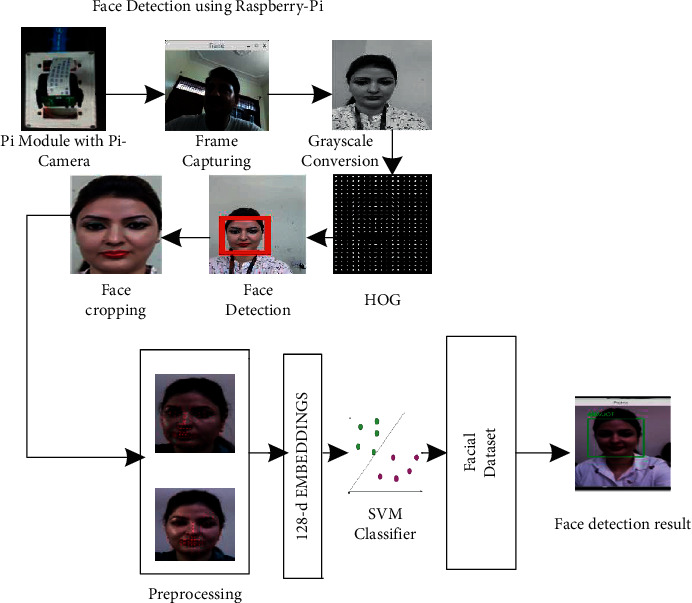
Face detection flow using Raspberry Pi.

Once the network has been trained, the next step is to recognize the face in real time. The detailed process of face recognition in video streams using Raspberry Pi is shown in Figure 18. This part of the methodology is a pre-trained model or deep learning model like the previous two stages of detector and embedder. Rather, this is SVM-based machine learning model for face recognition. In this stage, the CPU of the Raspberry Pi has been utilized to recognize the faces in real time.  Figure 19 shows the complete method of face recognition.

The entire process starts with the warming up of the camera sensor and the beginning of the video stream. A counter for counting frames per second has been initialized to benchmark. After capturing the frame from the video, it has been resized, and to detect the face in that frame a blob is also constructed. After the formation of blobs, the face can be detected, but before that weak detection needs to be filtered out and then extracts the ROI. The pointed ROI is then used to detect spatial dimensions to ensure correct recognition. After ensuring that the spatial dimensions are large enough and are more than the minimum probability, then finally the face blobs are constructed and passed through the embedder to generate a 128-d vector. SVM embedder is then finally predicted with the name and probability index.

**Figure 20:**
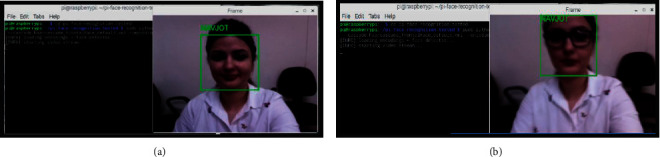
(a) Real-time face detection with name stamp. (b) Real-time face detection after wearing spectacles with a name stamp.

The face recognition results that are achieved from video implemented on Raspberry Pi in real time are shown in Figure 20. At this stage, the confidence has not been applied and only the exact match is displayed by displaying the name of the person after correct classification.

## 6. Conclusion

A facial recognition system is significant for the implementation of a vision-based system to detect abnormal activities and intruders in indoor and outdoor environment. The implementation of this kind of system with the dedicated hardware with better computation power is limitedly identified in previous studies. To overcome this challenge, in this study we have proposed vision-based intelligent architecture and systems to detect intruders through facial recognition and sensors with customized hardware. Vision- and sensor-based systems are customized and implemented support vector machine (SVM) along with a histogram of oriented gradients (HOG) in vision-based system. Even with a skewed image, the vision system can reach 99.9% accuracy for 20 different subjects, increasing security for intruder identification.

## Figures and Tables

**Table 1:** Comparative analysis of prior art.

Author	Function	Cloud	Accuracy	Customization
[14]	Raspberry Pi 3 Model B-based home security system was implemented with the Haar cascade algorithm and HOG	A cloud server is not implemented in this study	Accuracy of security system: Raspberry Pi 3: 100%; PIR sensor based: 76%	Customization is limited in this study for real-time implementation
[11]	Raspberry Pi with Yolo and Haar techniques are used to implement a human intrusion detection system	The cloud server is employed to store the detected intruder photos	83% accuracy for frontal face detection. 96% face or eye detection	Only Raspberry Pi is used for real-time implementation
[12]	MATLAB along with Raspberry Pi is used to detect emotions through speech	NA	Recognition efficiency: 85% in MATLAB and 95% on Raspberry Pi 3	The study implemented ready-made boards for the real-time implementation
[13]	The Haar cascade (face detection) and LBP (face recognition) algorithms are preferred for the real-time implementation of a system for monitoring the security with Raspberry Pi 2	The cloud server is used to store variations in motion	Accuracy is not discussed in this study	Raspberry Pi 2 is used for the implementation of the system
[15]	Integration of Viola–Jones algorithm, oriented FAST and rotated BRIEF (ORB) and SVM-based system is proposed for detecting suspects	The proposed classifier is stored and trained in the cloud	The algorithm's performance will improve with a better classifier	To investigate the performance of face detection algorithms on real-time video streams on the Raspberry Pi device

## Data Availability

For the data-related issue, kindly contact Navjot Rathour, er.rathour@gmail.com.
